# Type I ROP16 regulates retinal inflammatory responses during ocular toxoplasmosis

**DOI:** 10.1371/journal.pone.0214310

**Published:** 2019-03-22

**Authors:** Elise Rochet, Nicolas Argy, Valentin Greigert, Julie Brunet, Marcela Sabou, Luc Marcellin, Alejandra de-la-Torre, Arnaud Sauer, Ermanno Candolfi, Alexander W. Pfaff

**Affiliations:** 1 Institut de Parasitologie et Pathologie Tropicale, EA, Fédération de Médecine Translationnelle, Université de Strasbourg, Strasbourg, France; 2 Service de Parasitologie et Mycologie Médicale, Hôpitaux Universitaires de Strasbourg, Centre National de Référence de la Toxoplasmose, Pôle Sérologie, Strasbourg, France; 3 Service de Pathologie, Hôpitaux Universitaires de Strasbourg, Strasbourg, France; 4 Grupo NeURos, Unidad de Inmunología, Universidad del Rosario, Escuela de Medicina y Ciencias de la Salud, Bogotá, Colombia; 5 Service d’Ophtalmologie, Hôpitaux Universitaires de Strasbourg, Strasbourg, France; Centre National de la Recherche Scientifique, FRANCE

## Abstract

Ocular toxoplasmosis (OT), mostly retinochorioditis, is a major feature of infection with the protozoan parasite *Toxoplasma gondii*. The pathophysiology of this infection is still largely elusive; especially mouse models are not yet well developed. In contrast, numerous *in vitro* studies showed the highly *Toxoplasma* strain dependent nature of the host-parasite interactions. Some distinct polymorphic virulence factors were characterized, notably the rhoptry protein ROP16. Here, we studied the strain-dependent pathophysiology in our OT mouse model. Besides of two wild type strains of the canonical I (RH, virulent) and II (PRU, avirulent) types, we used genetically engineered parasites, RHΔROP16 and PRU ROP16-I, expressing the type I allele of this virulence factor. We analyzed retinal integrity, parasite proliferation and retinal expression of cytokines. PRU parasites behaved much more virulently in the presence of a type I ROP16. In contrast, knockout of ROP16 in the RH strain led to a decrease of intraocular proliferation, but no difference in retinal pathology. Cytokine quantification in aqueous humor showed strong production of Th1 and inflammatory markers following infection with the two strains containing the ROP16-I allele. In strong contrast, immunofluorescence images showed that actual expression of most cytokines in retinal cells is rapidly suppressed by type I strain infection, with or without the involvement of its homologous ROP16 allele. This demonstrates the particular immune privileged situation of the retina, which is also revealed by the fact that parasite proliferation is nearly exclusively observed outside the retina. In summary, we further developed a promising OT mouse model and demonstrated the specific pathology in retinal tissues.

## Introduction

*Toxoplasma gondii* is a ubiquitous apicomplexan parasite, infecting a third of the human population. In recent years, ocular toxoplasmosis (OT), mainly expressed as retinochoroididitis, have gained clinical attention, as survey studies revealed an unexpected prevalence of this affection of 1–2% in Europe and North America [1], most of which cannot be attributed to congenital infection [2]. While systemic infection is well explored, ocular infection remains poorly characterized despite its medical importance. Due to the specific immune privileged situation of the eye [3], the pathophysiological mechanisms are likely to be particular to this organ. The retina is organized in several alternate layers of nuclei and cell bodies of photoreceptors and other neuronal cells. Additionally, several types of glial cells help to maintain an anti-inflammatory environment and to preserve the integrity of the neuronal cells. In inflammatory conditions, the retinal architecture is usually visibly distorted, local glial cells activated and inflammatory leukocytes attracted [4]. We previously demonstrated this also for *T*. *gondii* infection [5]. In order to investigate the pathophysiological mechanisms, we previously characterized a murine OT model of intravitreal injection of the replicative tachyzoite stage in basic parasitological and immunological terms [6] and demonstrated the importance of the inflammatory Th17 pathway in infection with the relatively avirulent type II parasites [7]. We used and further developed this model in the present study.

Specifically, whether these findings are also valid for other *T*. *gondii* strains, has not been investigated. Therefore, we now expand our OT model to the comparison of different strains and the underlying strain-dependent factors. In the laboratory mouse model, differences between the canonical lineages existing in Europe and North America have long been known with type I strains rapidly killing mice, whereas type II strains, which are largely predominant in human infections, are much less virulent [8]. The discovery of highly variable and unusually virulent strains in immune competent humans in South America [9], where up to 17.7% of the population present retinal scars [10], adds complexity to this situation. The generally more severe OT clinical cases in South America are correlated with a completely contrasting ocular immune response [11], but the underlying strain-dependent virulence factors are not well explored in *T*. *gondii* infection studies [12].

Recently, products secreted from rhoptries and other apicomplexae-specific organelles were shown to hijack host-signaling pathways, which control the immune response, and therefore determine parasite virulence [13]. The rhoptry protein ROP16 was early identified as a strain specific key virulence factor [14]. This protein is injected into the host cell cytosol during parasite invasion, enters the nucleus and activates several transcription factors, such as STAT3, through its kinase activity [15]. The allele polymorphism of ROP16 is determined by a single amino acid difference [16] and plays a major role in the suppression of a protective Th1 type response by virulent type I but not by avirulent type II *Toxoplasma* strains [17]. This crucial role of ROP16 polymorphism was also found in South American human infections [18].

To characterize the role of ROP16 in the increased pathogenicity of type I strains, we first monitored the proliferation of wild and mutant type I and II strains in the eye. Then, we analyzed the expression of cytokines by retinal cells in response to infection with either wild type I and II strains or mutants including ROP16 deficient type I strain and type II strain expressing a heterologous type I allele of ROP16, in order to identify the influence of the virulent type I allele of ROP16 on intraocular parasite load and pathophysiology.

## Materials and methods

### Mice and parasites

Female inbred C57Bl/6J mice were purchased from Centre d’Elevage R. Janvier (Le Genest-Saint-Isle, France) and partially bred at our own animal housing facility (accreditation no A67-482-37). Animals were bred under specific pathogen-free conditions, according to national and local regulations.

Tachyzoites of the avirulent (type II) PRU and the virulent (type I) RH strain of *T*. *gondii* were obtained from the French Biological Resource Center Toxoplasma, Limoges and Reims, France. The PRU-ROP16-I strain was kindly provided by J. Saeij, UC Davis, USA and the RH-ΔROP16-I by J. Boothroyd, Stanford University, USA). Tachyzoites were maintained in human THP1 monocyte cultures or by weekly passages in Swiss-Webster mice. Preliminary assays showed similar parasite proliferation and cytokine signature of the RH and the RHΔKU80 strain, which was used to create the KO strain. RH parasites were thus used as control strain. The PRU-ROP16-I strain was created by inserting ROP16-I into a PRUΔHPT strain, but HPT was again inserted afterwards (J. Saeij, pers. comm.). Therefore, the wild-type PRU strain was used as control.

### Infection model

Mice at 4–8 weeks of age were infected through intravitreal injection with a solution of 2000 tachyzoites in 5μl of sterile PBS per eye. The solution was injected into the vitreous cavity (VC) of both eyes using 30-gauge needles (BD Microlance 3, Becton-Dickinson, Le Pont-de-Claix, France) on a 25μL syringe (Hamilton, Bonaduz, Switzerland) during general anesthesia with isoflurane (Forène, Abbott, Paris France) [6]. Age-matched control mice received an injection of 5μL of sterile PBS intravitreally. Four mice per group were euthanized at different time points post-infection by isofluorane overdose. Aqueous humor was collected by anterior chamber paracentesis (≥ 5 μl/eye), pooled and stored at -80°C until analysis. Eyes were enucleated and pools of whole eyes were collected and stored at -20°C or immediately preserved in 4% buffered formaldehyde for *Toxoplasma* specific DNA quantification or histopathology, respectively. Each experimental group consisted of four animals (8 eyes).

All experiments were performed in accordance with ARVO (Association for Research in Vision and Ophthalmology, USA) Statement for the Use of Animals in Ophthalmic and Vision Research, as well as with national and local restrictions. Bilateral injection allowed us to use fewer mice to obtain the necessary number of eyes per pool, while severe retinal pathology was never observed in the short time frame of our infections, according to our observations in previous studies. Our animal study, including bilateral injection, was approved by the local ethic committee (project no.AL/77/84/02/13).

### Quantification of parasite load

DNA was extracted from pools of eight whole eyes and eluted in 200μl of elution buffer, as previously described [19]. Total DNA concentration was determined by Nanodrop^®^. *Toxoplasma* specific DNA was quantified with real-time PCR on a LightCycler (Roche Diagnostics) using the Faststart DNA Master SYBR Green (Roche Diagnostics, Meylan, France), in 5 μl of DNA solution and a reaction volume of 20 μL, following the manufacturer’s recommendations. The repetitive *TgB1* gene of *T*. *gondii* was chosen as target, as it allows sensitive quantification even for avirulent strains [20]. The following primers were used: 5’-GGA GGA CTG GCA ACC TGG TGT CG CG-3’ and 5’-TTG TTT CAC CCG GAC CGT TTA GCA G-3’. The parasite load was calculated using external standards, extracted from known numbers of parasites, run in parallel, and calculated as parasites per μg DNA. The standard curve showed high degrees of linearity at least between 10^1^ and 10^6^ parasites per reaction. Viability of the parasites was assured for each batch by infecting cell cultures.

### Retinal histopathology

Formaldehyde fixed eyes were embedded in paraffin, serially cut into 5 μm sections and stained with haematoxylin and eosin, as previously described [21]. Sections were analyzed by light microscopy under 1000x magnification. Retinal pathology was assessed for distortion of retinal layers, vascular dilatation and presence of inflammatory cells, i.e. invasive monocytes and neutrophils, distinguished by their characteristic morphology, and their migration into the plexiform layers of the retina.

### Cytokine/ Chemokine profile in aqueous humor

The Bio-Plex Mouse 23-Plex Panel assay (Bio-Rad, Marne-la-Coquette, France) was used according to the manufacturer’s recommendations to measure cytokine and chemokine levels in aqueous humor. As each pool of aqueous humor from eight eyes consisted of ≥40μl, duplicates of 20μl samples were analyzed. The assay plate layout consisted in a standard series, one blank well and duplicates of 20 μL samples, diluted to 50 μL with BioPlex mouse serum diluent. Data were analyzed with Bio-Plex Manager TM software V1.1 (BioRad). The fold induction index was calculated as the ratio of cytokine concentration in infected mice and the mean concentration of PBS injected mice.

### Localization of retinal cytokine expression by immunofluorescence

Cryocut sections of whole eyes were prepared and processed for indirect immunofluorescence to detect the cells expressing different cytokines, as previously described [19]. Primary antibodies used are listed in S1 Table. The corresponding Alexa 488 or 546 conjugated secondary antibodies were purchased from Invitrogen.

### Statistical analysis

Quantification of ocular parasites was done with pools of 8 eyes, due to small volumes. Each experiment was repeated three times and the results displayed as means ± SD. Statistically significant differences between the strains on a given day p.i. were identified using one-way ANOVA test, followed by Bonferroni’s multiple comparison test (GraphPad). Quantification of cytokines was also done with pools of 8 eyes. Means ± standard deviations (SD) from duplicate measures are shown. One-way ANOVA test was used to calculate statistical significance between experimental groups at day 7 post-infection.

## Results

### Intraocular parasite multiplication

**Fig 1:**
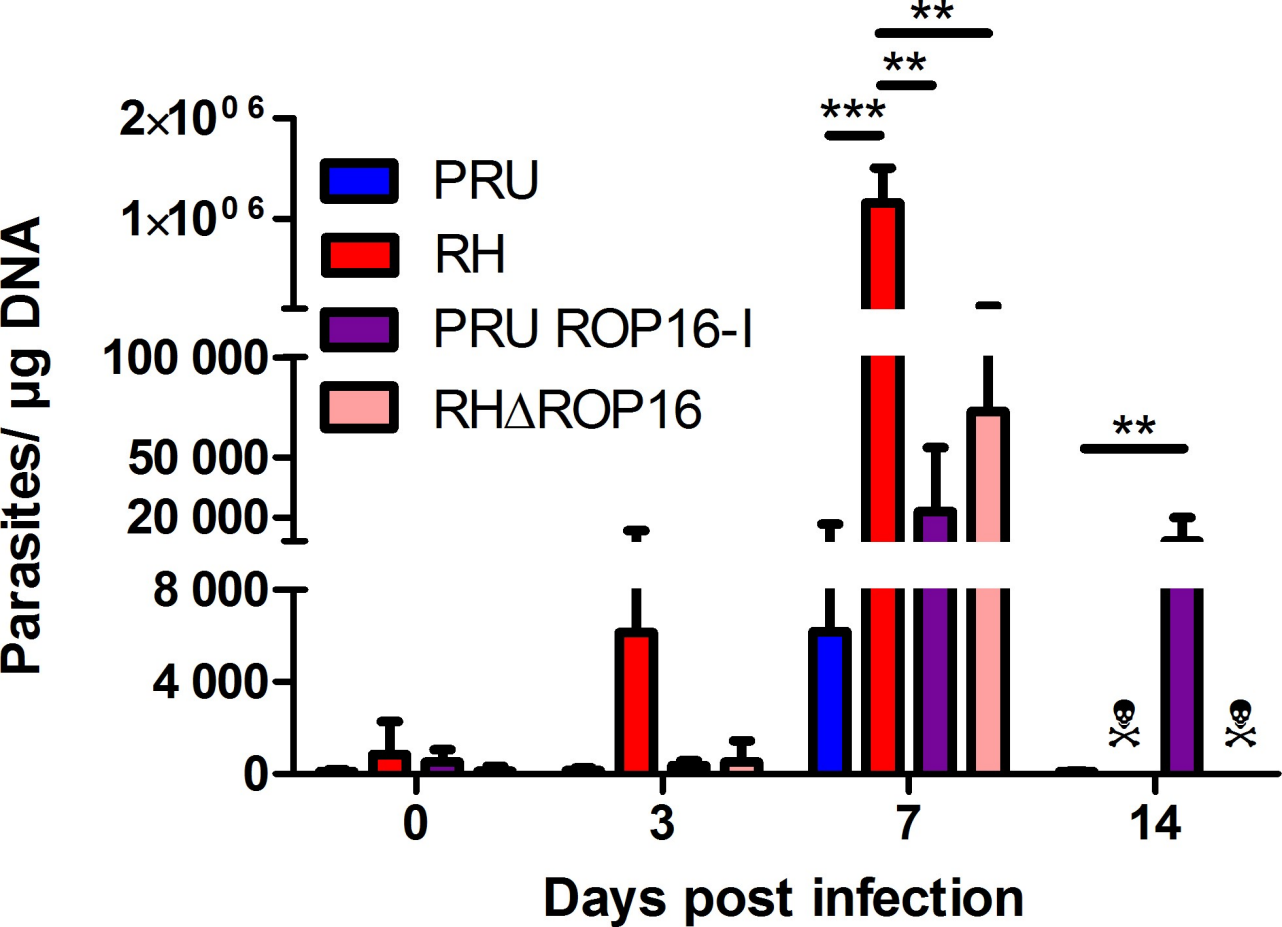
Ocular parasite load depends both on parental strain and ROP16 allele. C57BL/6 mice were infected by intraocular injection of 2000 *T*. *gondii* tachyzoites of different strains. The parasite load was determined by quantitative PCR on DNA extracted from whole eyes. Values shown are means ± SD of three independent experiments on pools of four mice (eight eyes). ☠ All mice infected with RH and RHΔROP16 strains died before 14 dpi. ** *P* < 0.01; *** *P* < 0.001.

To evaluate the virulence of different *Toxoplasma* strains and the particular role of ROP16, we injected tachyzoites into the eye. We first looked at parasite loads following infection (Fig 1). We observed completely distinct kinetics between the two parental strains. PRU strain parasite load increased moderately at 7 days post infection (dpi) and then decreased to very low levels at 14 dpi, while RH strain load increased steeply and rapidly. This intraocular injection rapidly led to fatal systemic infection, killing all RH infected mice between 7 and 14 dpi.

To evaluate the role of the virulence factor ROP16, we first evaluated the effect of a virulent ROP16-I allele in an avirulent type II *Toxoplasma* strain (PRU ROP16-I). The ocular parasite load sharply increased until 7 dpi, to a higher level than observed with the parental strain, but still fifty times lower than with RH infection. At 14 dpi, the parasite load diminished somewhat, but stayed at an elevated level, which was in complete contrast to the wild type PRU infection. However, all mice finally controlled infection and did not succumb to the PRU ROP16-I infection. Then, we determined the effect of the virulent ROP16-I allele in its native type I parasite by injecting RH parasites deficient of the *rop16* gene (RHΔROP16). The intraocular parasite load of this strain increased rapidly and to higher levels than with the PRU ROP16-I strain, but stayed significantly lower than with the wild type RH strain. Nevertheless, RHΔROP16 infection led to the death of all mice before 14 dpi.

### Retinal pathology

**Fig 2:**
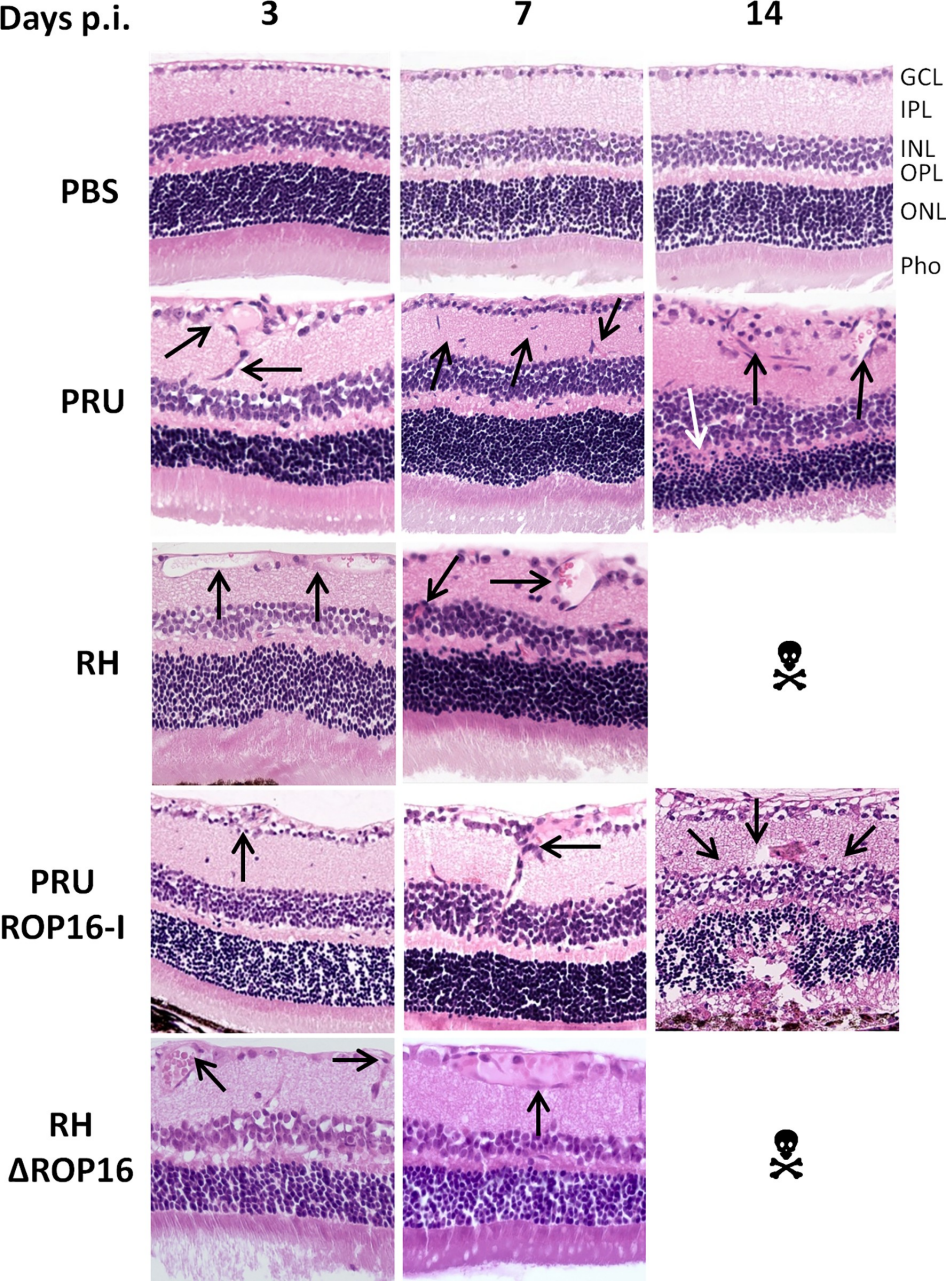
Retinal pathology following intraocular infection with different strains of *T*. *gondii* does not correlate with parasite load. C57BL/6 mice were infected by intraocular injection of 2000 *T*. *gondii* tachyzoites of different strains. Hematoxylin-eosin staining of retina sections. GCL ganglion cell layer; IPL inner plexiform layer; INR inner nuclear layer; OPL outer plexiform layer; ONL outer nuclear layer; Pho photoreceptor layer. ☠ All mice infected with RH and RHΔROP16 strains died before day 14. The arrows show the characteristic pathological features described in the text.

We next evaluated retinal sections on pathological consequences of infection with the different strains (Fig 2). Infection with PRU (type II) strain led to mild modifications of the nuclear layers and some cell invasion, mostly by neutrophils, which peaked at day 7. Moreover, retinal migration of mononuclear cells, monocytes or microglial cells was visible from 3 dpi. These modifications continued until 14 dpi and resulted in alterations in retinal architecture. Curiously, when the infection was carried out with the virulent RH strain, the retinal structure remained globally normal with only vessel vasodilatation in the ganglion cell layer and some cell migration observed.

The pathology observed following infection with the PRU ROP16-I strain was more severe than with either the parental PRU strain and the RH strain. Differences were already visible at 3 dpi, but became obvious by 7 dpi, with enhanced cell migration in the inner plexiform layer, but also in the outer plexiform layer. At 14 dpi, the two nuclear layers were distorted and showed less cell density. The photoreceptor layer showed equally cell loss. Additionally, inflammatory masses (black spots) were observed. Overall, the alterations observed here were considerably more important than that of the PRU wild-type strain at the same time. In contrast, upon infection with the RHΔROP16 strain, we observed, like in the RH parental strain, no notable difference in the architecture of the retina, but only some vasodilatation of the vessels in the ganglion cell layer.

These results show that the ROP16 allele has profound influence on retinal pathology, apparently independent on control of intraocular parasite multiplication.

### Ocular cytokine patterns

**Fig 3:**
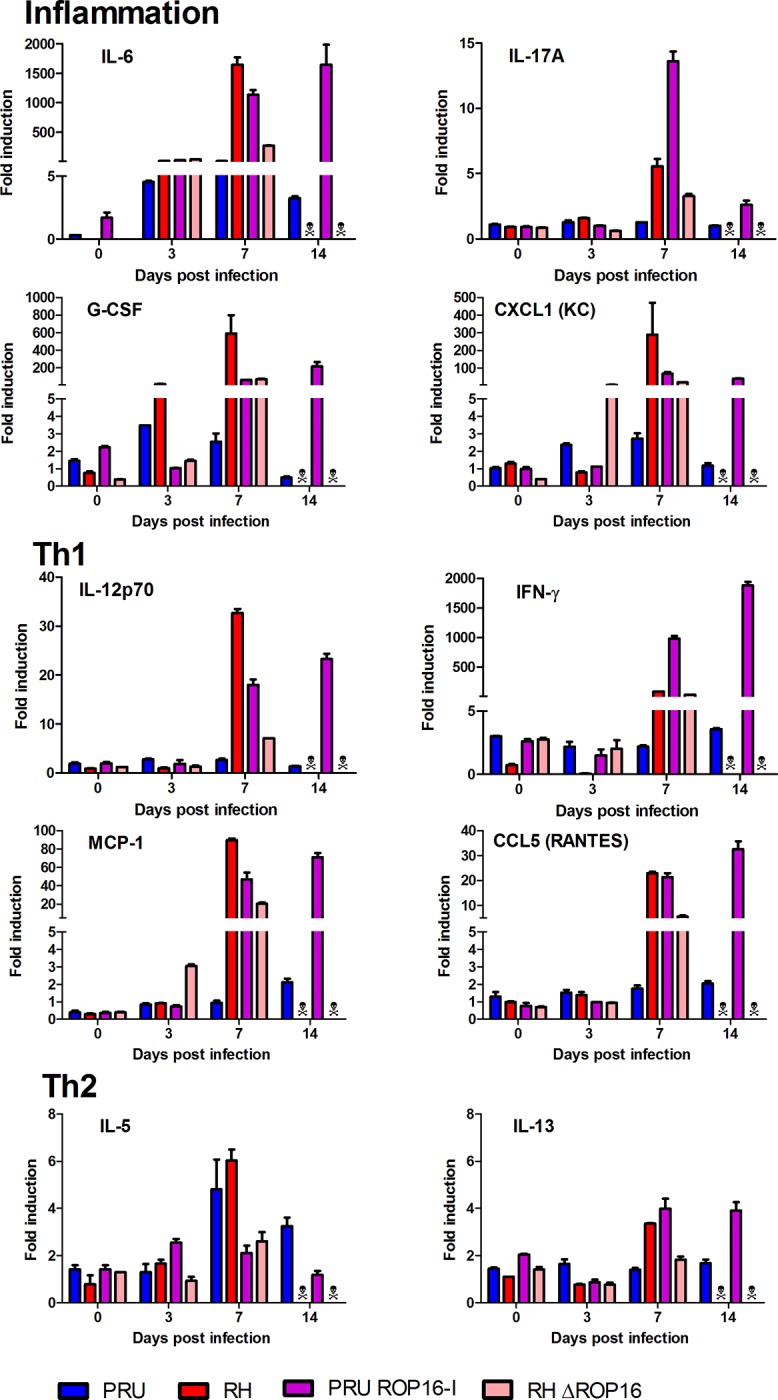
Cytokine levels in aqueous humor are determined by ROP16-I expression. C57BL/6 mice were infected by intraocular injection of 2000 *T*. *gondii* tachyzoites of different strains. Cytokine concentrations in aqueous humor were determined by BioPlex assays on pools of four mice (eight eyes) at the indicated time points. Values are means ± SD from duplicate measures, expressed as the fold increase of T. gondii-infected mice over PBS sham-injected mice. ☠ All mice infected with RH and RHΔROP16 strains died before day 14. Statistically significant differences between groups at 7 dpi are summarized in S2 Table.

The Th1 type immune response, characterized by the cytokines IFN-γ and IL-12, has long been acknowledged to have a protective function in *T*. *gondii* infection [22]. In contrast, our previous studies demonstrated the detrimental role of Th17 cytokines, characterized by the production of IL-17A, in OT, at least in infection with type II strains [7]. To evaluate these two cytokine groups in different strains, we quantified these cytokines in aqueous humor, using a BioPlex multiplex assay (Fig 3 and S1 Fig). PRU strain infection triggered a moderate inflammatory immune response, but no visible upregulation for the other cytokines. In contrast, with RH infection, Th1 and Th17 inflammatory responses were strongly upregulated at 7 dpi. Th2 cytokines were also upregulated, but only to moderate levels. Interestingly, PRU ROP16-I infection induced a marked increase in cytokine levels, compared to wild type PRU infection, and more similar to RH infection. Certain cytokine levels at 7 dpi were even higher than with RH infection. Remarkably, most cytokine levels were stable or even further increased at 14 dpi, again in contrast to wild type PRU infection. In contrast, the Th1 and Th17 cytokine levels of the RHΔROP16 strain followed the same increase at 7 dpi as observed with the wild type RH strain, but were throughout considerably lower. These results illustrate that the presence of the ROP16-I protein in an avirulent PRU strain considerably increased the local immune response, whereas the lack of this factor diminished it in the virulent RH strain.

**Fig 4:**
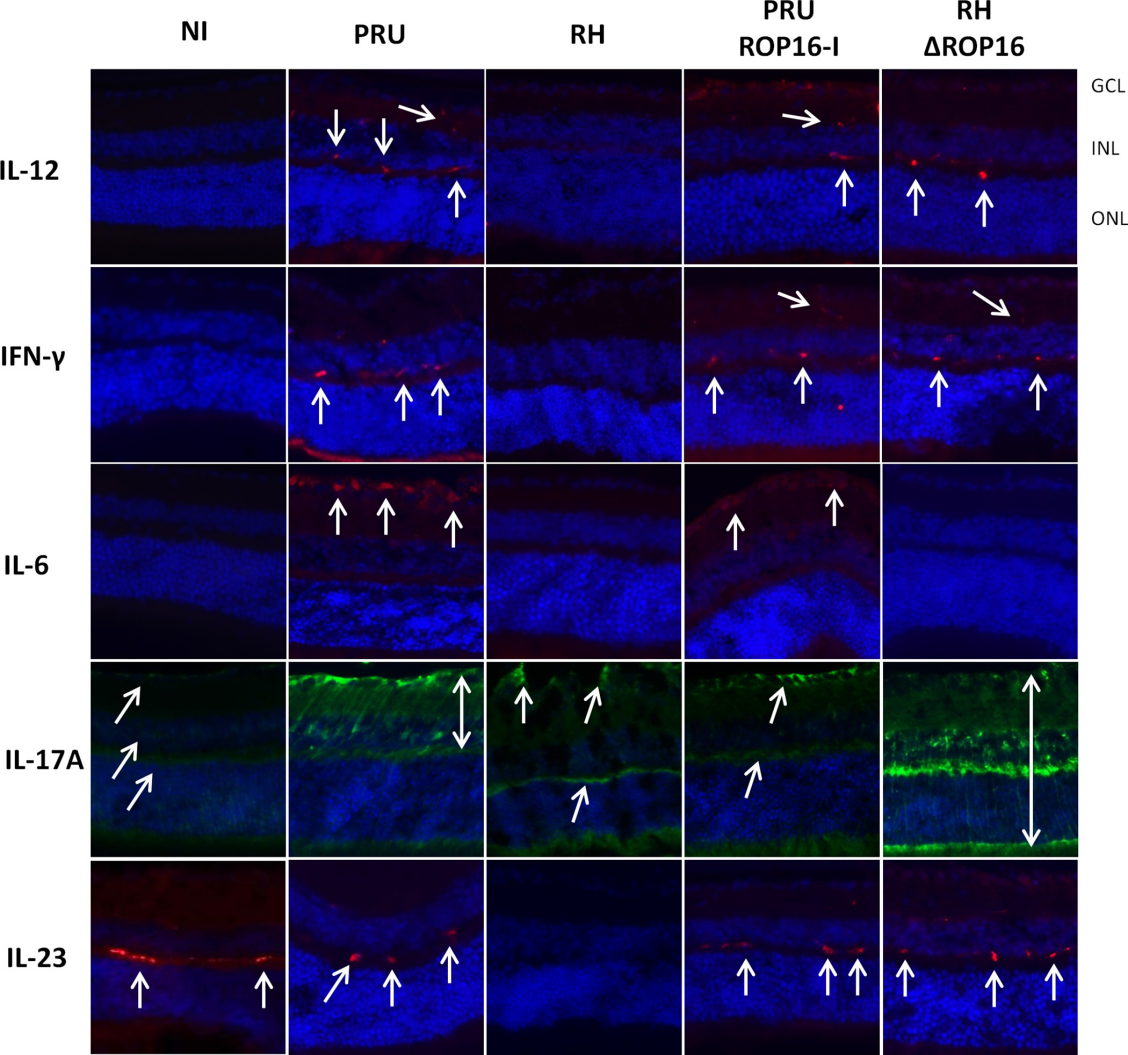
Retinal cytokine expression depends both on background strain and on the ROP16 allele. C57BL/6 mice were infected by intraocular injection of 2000 *T*. *gondii* tachyzoites of different strains. Retinal expression of Th1 and inflammatory cytokines was detected by immunoflurorescence 7 days after infection (arrows). GCL ganglion cell layer; INR inner nuclear layer; ONL outer nuclear layer.

Next, we looked at actual expression of these cytokines in retinal cells (Fig 4). Both IL-12 and IFN-γ were easily detected at 7 dpi in all strains with the notable exception of the RH parental strain. These two cytokines are principally localized to the inner and outer plexiform layers. We also looked at the inflammatory cytokines IL-6, IL-17A and IL-23: IL-6 was expressed in the ganglion cell layer following infection with the PRU parental and mutant strains, but absent with RH parental and knock-out strains. IL-17A was the only cytokine detected following infection with all strains, including RH. However, expression was more reduced with RH infection and, interestingly, also in the PRU ROP16-I strain. The expression of IL-17A was mainly detected in Muller cells, as we previously showed by vimentin co-localization in PRU infected retinas [7]. While IL-17A expression extended throughout the length of Muller cells following infection with the RHΔROP16 and PRU parental strains, expression in the RH and PRU ROP16-I infected mice seemed to be restricted to the endfeet region of Muller cells. This could suggest a perturbation of intracellular transport of IL-17 in the highly polarized Muller cells. Finally, IL-23 was the only cytokine clearly detected in non-infected mice. It was also detected in infected eyes, again except with RH parental strain infection. Expression of IL-23 was localized to the outer plexiform layer. These results suggest that type I *Toxoplasma* strains need the presence of their specific ROP16-I allele to regulate expression of Th1 and Th17 type cytokines, with the exception of IL-6. In contrast, in a type II strain, the ROP16-I allele seems to affect only expression of IL-17A in our setting.

**Fig 5:**
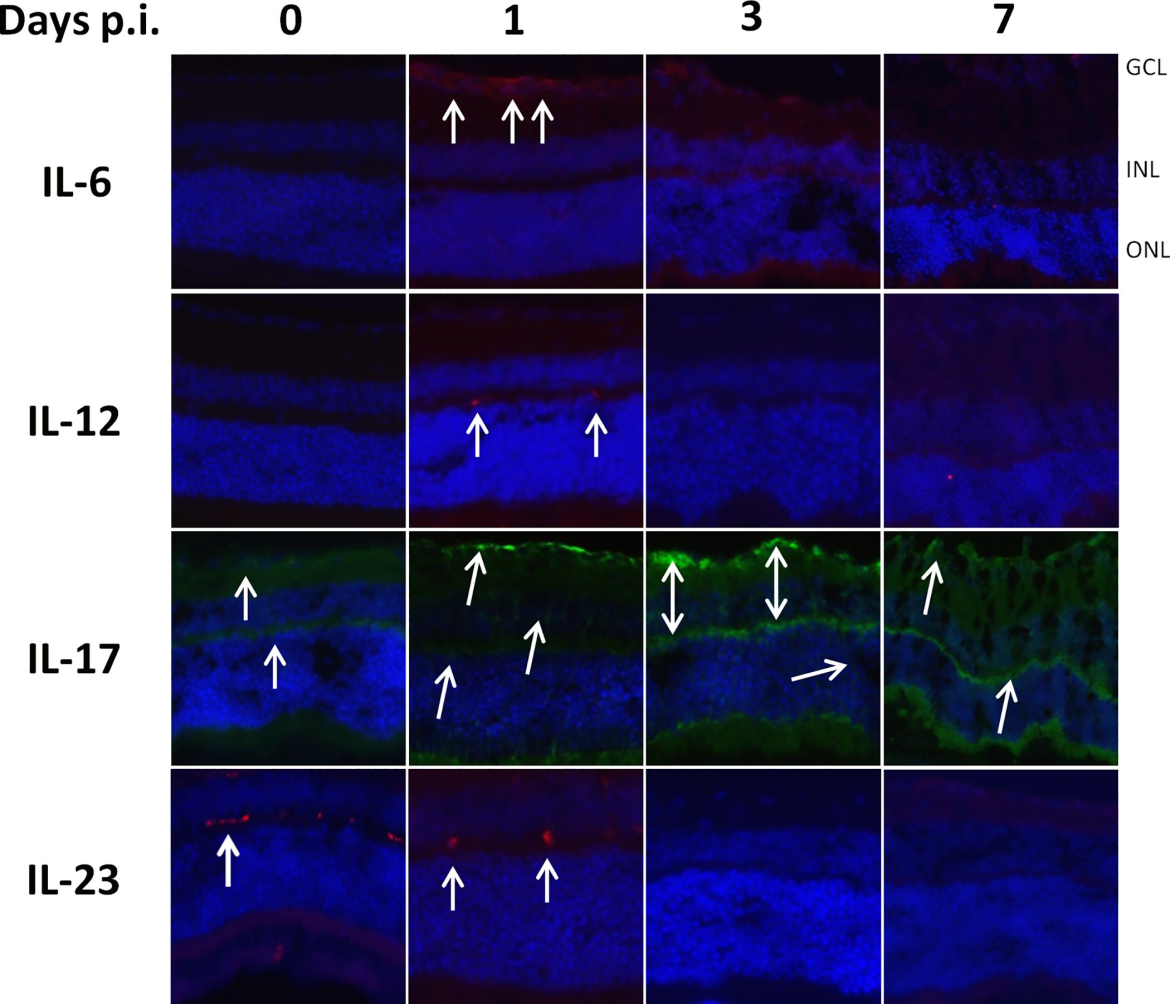
Early expression and subsequent loss of retinal cytokine production following intraocular infection with the RH strain of *T*. *gondii*. C57BL/6 mice were infected by intraocular injection of 2000 *T*. *gondii* tachyzoites of different strains. Cytokine producing cells were localized by immunofluorescence on retinal cryosections (arrows).

Regarding this notable absence of cytokine expression in RH strain infection at 7 dpi, we studied the early kinetics of cytokine expression upon RH infection (Fig 5). Interestingly, all cytokines were visibly expressed at 1 dpi., but absent thereafter. IL-17A stands out in this context, as expression developed more slowly and was at its maximum at day 3 p.i. At 7 dpi., IL-17A expression was visibly weaker. Interestingly, this expression kinetics of IL-17A was also observed with PRU ROP16-I infection (not shown), which suggests an involvement of ROP16 in the active suppression of IL-17A. These results show that inflammatory and Th1 cytokines are initially expressed following infection, before being actively suppressed by the type I *Toxoplasma* strain.

## Discussion

While type I strains of *T*. *gondii* are uniformly virulent in mice, type II strains display a chronic course of infection, despite differing at only few genetical loci [23]. In this study, we first observed that Type II infection could be controlled by the local immune response after a moderate proliferation activity during the first days post infection. However, this was accompanied by transient inflammatory processes, best visible by a massive influx of monocytes and neutrophils. In contrast, with the type I RH strain, we detected uncontrolled ocular parasite proliferation by PCR, and host death, indicating rapid systemic dissemination, but we observed little retinal pathology in microscopic observation of retinal sections at 7 dpi. This could be explained by the particular place in the immune regulation of the retina itself, closely linked to its immune privileged situation behind efficient cellular and immunological barriers, which is known to avoid overwhelming inflammatory reactions [24]. To further specify this particular role of the retina, it would be interesting to compare the kinetics of pathology in different organs, especially the brain, with different infection doses.

We aimed to decipher the role of the parasite strain and particularly of one of these factors, ROP16, on pathology and immune response in the particular ocular environment. Recently, the polymorphic rhoptry protein ROP16 was identified as major modulator centrally determining strain virulence [16, 17]. The fact that the majority of ROP16 nucleotide sequences from Colombian patients with ocular toxoplasmosis (80%) belonged to the group of mouse-virulent strains ROP16-I allele [18] demonstrates its relevance for human pathology. Therefore, we focused on the role of the ROP16 type I allele separately from the background genes, in our *in vivo* model. The lower parasite counts in the RHΔROP16 and the considerably elevated ocular parasite load in the transgenic PRU strain expressing the heterologous ROP16-I allele, compared to the two parental strains, strongly suggests a role of the ROP16-I allele as a virulence factor activating efficient immune evasion mechanisms. This virulence enhancing effect of ROP16-I is also clearly visible in the much more severe retinal disorganization in the PRU ROP16-I strain. A previous study with the same transgenic PRU ROP16-I strain observed a nearly complete absence of parasite proliferation in the intestine after oral infection [25]. Another work found, in contrast to our results, enhanced parasite loads in multiple organs with the type I ΔROP16 strain [26]. Interestingly, while this difference was highly significant in spleen, liver and lung, it was not in brain, another immunoprivileged organ with important similarities with the eye. This could point to crucial differences of systemic and ocular infection. Specifically, Butcher *et al*. linked limited *Toxoplasma* proliferation to ROP16-I dependent activation of host arginase production. This is consistent with our observation that the transgenic PRU ROP16-I strain does not cause host death, despite of high ocular parasite proliferation. In view of our results, the arginase pathway might be differently regulated in the ocular environment. In contrast, the similar weak retinal pathology in wild type and knockout RH strains argues against an active role of ROP16-I in the pathological process itself.

The outcome of *Toxoplasma* infection is determined by the induced cytokine response. We looked both at cytokines present in aqueous humor and actual cytokine production by retinal cells and observed different kinetics. Cytokine levels in the aqueous humor were largely determined by the presence of the ROP16-I allele in the infecting parasite strain. The two strains containing the ROP16-I allele induced high levels of most cytokines, largely correlating with parasite loads. Seven days post-infection, high levels of inflammatory and Th1 cytokines are detected in the aqueous humor. The PRU ROP16-I strain allowed us to follow the infection beyond seven days, contrary to the RH strain. Interestingly, most cytokine levels were stable between days 7 and 14, in contrast to wild type PRU infection, which again parallels the parasite loads. On the other hand, the mutant PRU strain could finally be controlled and did not induce mortality. Consequently, there seems to be an as yet unexplained interplay between the ROP16-I allele which dramatically increases parasite numbers and immune response, and a dampening effect of the PRU background, in which the host is capable to finally control parasite proliferation and avoid lethal overreaction.

In contrast, retinal cytokine expression, as assessed by immunofluorescence, is initially induced, but then rapidly and strain-dependently suppressed. More precisely, suppression of IL-17A expression seems to be entirely due to the virulent ROP16 allele, while the other cytokines are suppressed by type I ROP16 only within the type I strain. It would now be interesting to look on ROP16 induced cell-type specific reactions, such as STAT3 activation, a known mediator of ROP16 action [15]. Our result that the virulent strain suppressed local cytokine production more efficiently than avirulent strain could explain the absence of visible retinal pathology in RH infection, whereas PRU infection causes moderate, probably immune mediated pathology. This recalls our previous results on human OT, which also suggested an immune induced pathology in type II infections in European patients, whereas the ocular immune response in South American patients were down-regulated and the pathology seemed to be induced by uncontrolled parasite proliferation [11]. Obviously, the short observation period due to rapid host death in type I infections does not allow to monitor the full ocular disease progression. Concordantly, *in vitro* studies showed that virulent but not avirulent strains down-regulate Th1 cytokine production [27]. This seems to be in contrast to the much stronger production of IFN-γ in type I infected mice [28]. More detailed studies suggested that overproduction of inflammatory cytokines *in vivo* is probably secondary to uncontrolled parasite growth [17]. In light of these results, RH specific suppression in our study could be just due to higher parasite loads with this strain. However, this suppression was visible as early as 3 dpi, where parasite load in RH infection was still quite low, whereas suppression was not observed at 7 dpi in the RHΔROP16 and PRU ROP16-I strains, with considerably higher parasite loads. To fully answer this question, it would be again interesting to compare the outcome of various infection doses. The coincidence of rather low local cytokine production, as assessed by immunofluorescence, and a nearly complete lack of detectable parasite proliferation or even persistence within the retina could be due to a common mechanism of immunosuppression of retinal cells and inhibition of *Toxoplasma* proliferation. Retinal production of indoleamine 2,3 dioxygenase (IDO), which consumes the available tryptophan, has been demonstrated [29] also upon *T*. *gondii* infection, and shown *in vitro* to restrict parasite growth [30]. There are certainly several of these mechanisms at play, and further studies could bring valuable insights in retinal anti-parasitic pathways. The reason for the contrasting cytokine patterns in aqueous humor has still to be elucidated. It could be explained by invading cells or a less suppressed production outside the retina.

The immunofluorescence study also allowed to localize retinal cytokine production. For the Th1 cytokines IL-12 and IFN-γ, their localization to the plexiform layers and microscopical aspect of the producing cells suggest them to be microglial cells. Brain microglial cells are well known producers especially of IL-12, which was again recently shown for the related apicomplexan parasite *Neospora caninum* [31]. Further studies could confirm this by immunophenotyping and distinguishing microglia from invading monocytes. Expression of IL-17A was observed in Muller cells, as in our previous studies [7], but seems also to be expressed in other cell types. Muller cells could be a central cell type in the development of OT, as they were recently shown to harbor tissue cysts, unlike microglial cells and astrocytes [32]. Our ocular model is particularly suitable to investigate such development of an immune response, due to confinement of a complex immune network to this small immunoprivileged organ. It would be interesting to follow the spatial and temporal kinetics of parasite conversion into bradyzoites and cysts following such intraocular injection.

In summary, we introduced a model to compare ocular pathology and parasite proliferation for *Toxoplasma* strains of different virulence. These results show different pathological consequences of ocular, compared to systemic, infection, demonstrating that the specific immunosuppressive ocular environment is maintained upon *T*. *gondii* infection. Some of our findings, such as the differences between cytokine levels in aqueous humor and cellular cytokine production, or the absence of severe pathology in RH-infected retinas, show the complex situation of the eye and should be further examined. Our results on the central importance of the ROP16 alleles pave the way for further mechanistic studies on the effect of this virulence factor. Finally, these studies should also be extended to the highly variable and often highly pathogenic South American strains and compared with sequence analysis and comparison of selected virulence factors, notably ROP16.

## Supporting information

S1 Fig Bioplex measurement of cytokine levels in aqueous humor: Cytokines not shown in Fig 3. C57BL/6 mice were infected by intraocular injection of 2000 *T*. *gondii* tachyzoites of different strains. Cytokine concentrations in aqueous humor were determined by BioPlex assays on pools of four mice (eight eyes) at the indicated time points. Values are means ± SD from duplicate measures, expressed as the fold increase of T. gondii-infected mice over PBS sham-injected mice. ☠ All mice infected with RH and RHΔROP16 strains died before day 14.
(TIF)

S1 File Data set for Figs 1 and 3 and S1 Fig. (XLSX)

S1 Table Primary antibodies used for immunofluorescence. ^a^ SC: Santa Cruz Biotechnology; Ab: Abcam.
(DOCX)

S2 Table One-way ANOVA test of differences of cytokine levels between groups at 7 dpi in Fig 3. Shown are mean differences between groups, *P* values (ns not significant, * <0.05, ** <0.01, *** < 0.001, **** < 0.0001) and 95% confidence intervals, as calculated by GraphPad.
(XLSX)
