# Supplementary material for: Type I ROP16 regulates retinal inflammatory responses during ocular toxoplasmosis
Source: PLoS One. 2019 Mar 22;14(3):e0214310. doi: 10.1371/journal.pone.0214310 (PMC6430381; doi:10.1371/journal.pone.0214310)
Supplement: S1 Table — a SC: Santa Cruz Biotechnology; Ab: Abcam. (DOCX) [file pone.0214310.s003.docx]

**S1 Table. Primary antibodies used for immunofluorescence**

| **Target** | **Species** | **Source^a^** | **Clone/Reference** |
| --- | --- | --- | --- |
| IFN-γ | Rat monoclonal | SC | R4-6A2 |
| IL-12p70 | Rat monoclonal | SC | EQ-7 |
| IL-6 | Rabbit polyclonal | Ab | ab6672 |
| IL-17 | Rabbit polyclonal | SC | H-132 |
| IL-23 | Rabbit polyclonal | SC | H-113 |
